# Independent Evolution of Sex Chromosomes in Eublepharid Geckos, A Lineage with Environmental and Genotypic Sex Determination

**DOI:** 10.3390/life10120342

**Published:** 2020-12-10

**Authors:** Eleonora Pensabene, Lukáš Kratochvíl, Michail Rovatsos

**Affiliations:** Department of Ecology, Faculty of Science, Charles University, 12844 Prague, Czech Republic; pensabee@natur.cuni.cz (E.P.); lukas.kratochvil@natur.cuni.cz (L.K.)

**Keywords:** Gekkota, reptiles, DNA-seq, sex chromosomes, sex determination, qPCR

## Abstract

Geckos demonstrate a remarkable variability in sex determination systems, but our limited knowledge prohibits accurate conclusions on the evolution of sex determination in this group. Eyelid geckos (Eublepharidae) are of particular interest, as they encompass species with both environmental and genotypic sex determination. We identified for the first time the X-specific gene content in the Yucatán banded gecko, *Coleonyx elegans*, possessing X_1_X_1_X_2_X_2_/X_1_X_2_Y multiple sex chromosomes by comparative genome coverage analysis between sexes. The X-specific gene content of *Coleonyx elegans* was revealed to be partially homologous to genomic regions linked to the chicken autosomes 1, 6 and 11. A qPCR-based test was applied to validate a subset of X-specific genes by comparing the difference in gene copy numbers between sexes, and to explore the homology of sex chromosomes across eleven eublepharid, two phyllodactylid and one sphaerodactylid species. Homologous sex chromosomes are shared between *Coleonyx elegans* and *Coleonyx mitratus*, two species diverged approximately 34 million years ago, but not with other tested species. As far as we know, the X-specific gene content of *Coleonyx elegans / Coleonyx mitratus* was never involved in the sex chromosomes of other gecko lineages, indicating that the sex chromosomes in this clade of eublepharid geckos evolved independently.

## 1. Introduction

Sex determination is the vital process that determines whether an individual will develop to a male or a female. Despite its importance in numerous ecological and evolutionary processes, organisms have not converged to a single mechanism of sex determination. Amniotes, the clade of vertebrates comprising sauropsids (reptiles and birds) and mammals, demonstrate two major systems of sex determination: the genotypic sex determination (GSD), where the sex is determined by sex-specific genetic factors linked to sex chromosomes, and the environmental sex determination (ESD), where the sex is influenced by environmental factors, most commonly temperature (temperature-dependent sex determination—TSD), during a sensitive period of embryonic development and there are no sex-specific differences in genotypes [1,2]. In addition to sexual reproduction, few reptilian species can reproduce asexually, mostly by hybridogenesis, obligate or facultative parthenogenesis [3,4].

The ancestral ESD hypothesis suggests that the common ancestor of amniotes had ESD and that sex chromosomes evolved independently in distant lineages, often from the ancestral ESD [5,6]. The major support for this hypothesis comes from phylogenetic reconstruction of the sex determination systems in reptiles indicating that transitions from ESD to GSD are common, but transitions in the opposite directions are rare. The hypothesis expects that once evolved, GSD seems to be evolutionary stable in a long term, acting as an “evolutionary trap” and predicts that amniote evolutionary lineages phylogenetically separated by an ESD lineage should have non-homologous sex chromosomes [5,6,7]. An alternative hypothesis suggests that GSD was the ancestral sex determination system in amniotes. Currently the most popular formulation of the ancestral GSD hypothesis suggests that the common ancestor of amniotes had a “super-sex chromosome” with genomic content homologous to both the Z chromosome of birds and the X chromosome of viviparous mammals. During the time, chromosome rearrangements fragmented the ancestral “super-sex chromosome”, but some of the emerged chromosomes still maintain the role as sex chromosomes in extant, phylogenetically distant lineages of amniotes. The major support for this hypothesis originates from the partial similarity of the sex chromosome gene content between birds, monotremes and the gecko *Gekko hokouensis* [8,9,10,11,12,13,14], as well as between viviparous mammals and lacertid lizards [12]. The hypothesis of the ancestral GSD expects that ESD evolved multiple times within amniotes and that ESD can be phylogenetically nested within a paraphyletic group whose members share homologous GSD. The major criticism of the “super-sex chromosome” hypothesis concerns the possibility of independent co-option of genomic regions, with genes involved in gonad differentiation (such as *amh*, *ar*, *dmrt1* and *sox3*), for the role of sex chromosomes [12,15,16].

Geckos (Gekkota) are the sister group to all other squamate reptiles, except for dibamids [17,18] and therefore, a phylogenetically informative lineage for the reconstruction of the evolution of sex determination in amniotes. In contrast to mammals, birds and many squamate lineages such as lacertids, caenophidian snakes, iguanas, varanids and skinks (reviewed in [19]), geckos demonstrate an extensive variability in sex determination including ESD as well as male and female heterogamety [5,20,21]. It is possible that from unknown reasons geckos during their evolutionary history exhibit for an amniote lineage unusually high frequency of turnovers in sex determination systems. Nevertheless, the ancestral ESD hypothesis in amniotes proposes another explanation: it predicts that the gecko ancestor possess ESD and sex chromosomes evolved independently among geckos from ESD several times, which creates the variability in sex determination. The same situation was reconstructed in turtles, another amniote group with a large variability in sex determination [6]. In any case, geckos are an excellent group for testing general hypotheses on sex determination. Molecular cytogenetics and next generation sequencing methodologies (e.g., DNAseq, RNAseq, RADseq) allow us to gradually start resolving the puzzle of the gecko variability in sex determination [7,16,22,23,24], but data are still scarce in this old, highly diversified lineage encompassing over 2000 currently recognized species [25]. Up to now, the eyelid geckos (family Eublepharidae), the gecko group with phylogenetically important position among squamates, was largely neglected in the application of modern sequencing approaches for the study of sex determination.

The family Eublepharidae is a group of approximately 40 species classified into six genera with a wide, but scattered distribution in Africa, Asia and Central and North America [25]. Among geckos, eublepharids possess several putative primitive morphological characters such as movable eyelids, soft, parchment-like eggshells and the lack of adhesive toepads. Traditionally, in phylogenies based on morphology, eublepharids were a primitive group sister to all other extant gekkotan lineages [26,27]. On the contrary, the molecular phylogenies put them sister to the monophylum of the families Sphaerodactylidae, Phyllodactylidae and Gekkonidae, with the monophylum of the families Pygopodidae, Carphodactylidae and Diplodactylidae being considered as their first outgroup [17,28,29,30]. Nevertheless, the recent genome-wide examination of the squamate phylogeny demonstrated that the true placement of eublepharids remains unresolved [31,32]. Eublepharids have a notable variability in sex determination systems. *Eublepharis macularius* and *Hemitheconyx caudicinctus* have ESD, which was documented by a strong dependence of hatchlings´ sex ratios on incubation temperatures. Females are produced at both low and very high incubation temperatures, while males occur mainly at intermediate temperatures [33,34,35,36,37,38,39,40]. GSD has been documented in four species of the genus *Coleonyx* (*Coleonyx brevis, Coleonyx variegatus, Coleonyx mitratus, Coleonyx elegans*) by balanced sex ratios at a variety of incubation temperatures [39,40,41]. Furthermore, X_1_X_1_X_2_X_2_/X_1_X_2_Y system of multiple neo-sex chromosomes evolved likely by a fusion of the ancestral Y chromosome with an autosome was identified in *Coleonyx elegans* by conventional and molecular cytogenetic methods [42]. The Y chromosome of this species is easily recognizable, as it is the only bi-armed chromosome in the karyotype. Such neo-Y chromosome was not found in *Coleonyx brevis* and *Coleonyx variegatus* [42]. The sex determination system of other eublepharid species remains uncertain, as the incubation experiments conducted, e.g., in *Aeluroscalabotes felinus* and species of the genus *Goniurosaurus* were inconclusive, as these studies either had small sample size or did not cover a wide range of temperatures (reviewed in [20,43,44]).

In the current project, we aim to shed light on the gene content and the homology of sex chromosomes in eublepharid geckos, a poorly studied and phylogenetically informative lineage, and to expand our knowledge on the reconstruction of the evolution of sex determination systems in amniotes. In this context, we sequenced by Illumina HiSeq2500 platform, the genomes of both sexes of the Yucatán banded gecko *Coleonyx elegans*, the only eublepharid species with documented sex chromosomes. We applied a comparative genome coverage analysis to reveal X-specific genes. The candidate X-specific genes were validated by quantitative real-time PCR (qPCR). Later, the same qPCR method was used to explore the homology of X-specific genes of *Coleonyx elegans,* across 11 additional species of eublepharid geckos (Table S1, in the Supplementary Materials), covering all six extant genera, as well as in three species from the families Phyllodactylidae and Sphaerodactylidae.

## 2. Results

### 2.1. DNA-seq and Coverage Analysis

**Figure 1:**
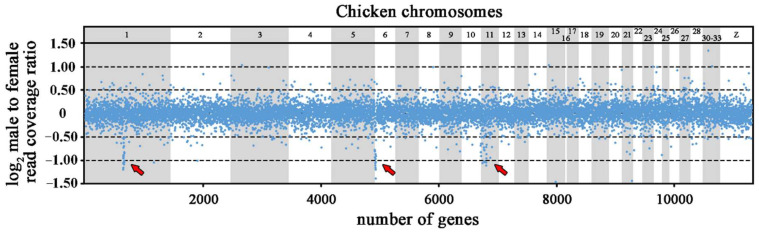
Log_2_-transformed male to female ratios of DNA-seq read coverage per gene in *Coleonyx elegans*. The X-specific genes have half male to female read coverage ratio than autosomal and pseudoautosomal genes, i.e., log_2_-transformed ratios about −1.0. The position of gene orthologs in chicken chromosomes is presented. X-specific genes with homologs linked to GGA1, GGA6 and GGA11 chromosomes are indicated by arrows.

The trimming and the quality filtering of the raw Illumina reads resulted in 422 million reads from the genome of the male and 432 million reads from the genome of the female of *Coleonyx elegans*. The trimmed reads were mapped independently with Geneious Prime [45] to a reference dataset of exons extracted from the *Gekko japonicus* genome project [46]. After removal of the assemblies of exons with 3-fold difference from the mode coverage, the final dataset consisted of 18.691 genes with an average genome coverage of 33x in the male and 32x in the female (Table S2, in the Supplementary Materials). Subsequently, we calculated the ratio of male to female (m/f) read coverage for each gene normalized for the total number of assembled reads per individual. The comparative read coverage analysis revealed 201 genes with the m/f ratio expected for the X-specific genes, i.e., within the interval 0.35–0.65 (Table S2). Among the 201 putative X-specific genes, 83 genes had known homology to chicken (*Gallus gallus* - GGA) genes. Specifically, 24 genes have homologs linked to 3.5 Mbp region of chicken chromosome 1 (GGA1), 14 genes to 2.5 Mbp region of chicken chromosome 6 (GGA6), 19 genes to 3.2 Mbp region of chicken chromosome 11 (GGA11). The orthologs of 26 genes assigned as putatively X-specific in *Coleonyx elegans* are scattered across 15 other chicken chromosomes (Figure 1; Table S2).

### 2.2. Validation of X-Specific Genes by qPCR in C. elegans and Test of Sex Chromosome Homology in Other Geckos

We applied the qPCR methodology to compare the difference in gene copies between males and females and thus validate their X-specificity in *Coleonyx elegans*. We designed primers from four genes with homologs to GGA1 (*dusp16, mansc4, ttc38, vwf*), four genes with homologs to GGA6 (*bms1, lrit1, lrit2, sncg*) and two genes with homologs linked to GGA11 (*ddx28, osgin1*). In addition, the autosomal genes *mecom* and *noct* were used for normalization of the qPCR values and as autosomal control, respectively. The qPCR test revealed that all 10 tested genes with orthologs linked to GGA1, GGA6 and GGA11 are X-specific in *Coleonyx elegans* (Table S3, in the Supplementary Materials).

**Figure 2:**
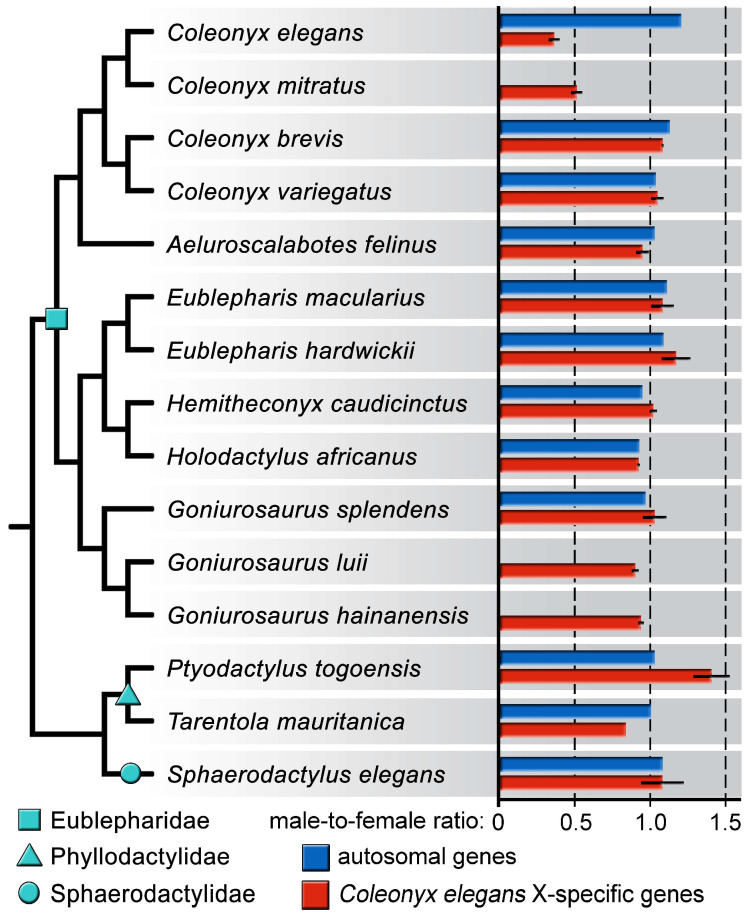
Average relative gene dose ratios between sexes of autosomal (blue bars) and X-specific genes (red bars), identified from the comparative genome coverage analysis in *Coleonyx elegans* and tested by qPCR across 12 eublepharid, two phyllodactylid and one sphaerodactylid species. Among all tested species of geckos, only *Coleonyx elegans* and *Coleonyx mitratus* share the same X-specific genes. Phylogeny follows [17]. All data are presented in Table S3.

Six X-specific genes of *Coleonyx elegans* (*bms1, ddx28, dusp16, lrit1, lrit2, mansc4*) were tested by qPCR across 14 other species of eublepharid, phyllodactylid and sphaerodactylid geckos to explore if they share homologous XX/XY sex determination system. The qPCR test revealed that the six tested genes are also X-specific in *Coleonyx mitratus*, but not in the other tested species of geckos, including the congeneric *Coleonyx brevis* and *Coleonyx variegatus* (Figure 2; Table S3).

## 3. Discussion

Our study identified for the first time the X-specific gene content of *Coleonyx elegans* by comparative read depth analysis between a male and a female genome, sequenced by Illumina HiSeq2500 platform. The X-specific region in this species seems to be relatively small, consisting of 201 genes, with orthologous syntenic blocks linked predominantly to parts of three chicken chromosomes (GGA1, GGA6 and GGA11). The same syntenic blocks seem to be X-specific also in *Coleonyx mitratus* as revealed by qPCR, indicating that XX/XY sex determination system emerged in their common ancestor approximately 34 million years ago, which is the estimated age of the basal split of the two species [28,29,30].

The karyotype of *Coleonyx elegans* consists of 2n = 32 chromosomes in females, but 2n = 31 chromosomes in males [42]. The same study described the derived system of X_1_X_1_X_2_X_2_/X_1_X_2_Y multiple sex chromosomes in *Coleonyx elegans* by chromosome painting with probe specific to the Y chromosome and male meiotic chromosome spreads, documenting a trivalent formed by the X_1_, X_2_ and Y chromosomes. It seems that the Y chromosome of *Coleonyx elegans* evolved via a Robertsonian fusion of the ancestral Y chromosome with an autosome. Notably, this neo-Y chromosome is not present in *C. variegatus* and *C. brevis* (2n = 32 in both sexes) [42], but cytogenetic evidence is missing in *Coleonyx mitratus*. Chromosome painting with probe specific for the neo-Y chromosome revealed a uniform hybridization on both the X_1_ and X_2_ chromosomes in *Coleonyx elegans* [42], indicating that the large Y chromosome is not particularly degenerated, which corresponds to the results based on the coverage analysis.

In amniotes, sex chromosomes often seem to evolve from genomic regions with genes involved in the gonad development and differentiation [10,15,16]. The syntenic block homologous with GGA1 has been previously reported as being a part of sex chromosomes in skinks [19], also under male heterogamety. The syntenic block homologous with GGA11 was previously reported as a part of the sex chromosomes in monotremes, where it is probably not connected to sex determination but represents a later added region [47], but not in any reptile lineage. As far as known, the GGA6 syntenic block is not a part of sex chromosomes in any reptile species either. 

Closer comparison of the *Coleonyx elegans* and the *Scincus scincus* [19] X-specific gene content revealed a small overlap. GGA1, GGA6 and GGA11 syntenic blocks are enriched in genes involved in gonad development and differentiation or with ectopic expression leading to sex reversals (Table S4, in the Supplementary Materials). Such genes, e.g., *ep300* [48], *fgf9* [49], *fgfr2* [50] and *sox10* [51,52], can be considered as candidate sex-determining genes in the genus *Coleonyx*. Nevertheless, the comparative gene coverage analysis identifies only genes from the X-specific region degenerated on the Y, while X- and Y-linked gametologs with high sequence similarity and genes exclusively linked to the Y and X are not revealed. Further studies are required to identify the sex-determining gene, pseudoautosomal regions and the ancestral and newly added sex chromosome regions in *Coleonyx elegans.*

All other tested eublepharid, phyllodactylid and sphaerodactylid species do not seem to share sex chromosomes homologous to those of *Coleonyx elegans* and *Coleonyx mitratus*. Such outcome was expected for *Eublepharis macularius* and *Hemitheconyx caudicinctus*, which have well-documented ESD, and therefore, sex chromosomes should be absent. In the genus *Goniurosaurus*, GSD was previously suggested for *Goniurosaurus araneus, Goniurosaurus luii* and *Goniurosaurus lichtenfelderi* [44,53], and ESD for *Goniurosaurus orientalis*, *Goniurosaurus splendens* and *Goniurosaurus kuroiwae* [53] based on the hatchling sex ratio, but these findings are inconclusive due to either small sample size or limited range of incubation temperatures [20]. Surprisingly, *Coleonyx elegans* and *Coleonyx mitratus* do not share the X-specific regions with the congeneric *Coleonyx brevis* and *Coleonyx variegatus,* despite all four species have GSD [39,40,41]. The current analysis cannot decide whether the X-specific genomic regions revealed by coverage analysis (i.e., regions orthologous to GGA1, GGA6, GGA11) in *Coleonyx mitratus* and *Coleonyx elegans* are from the degenerated neo-parts of the Y, or from the ancestral Y region. Future studies should determine the gene content of sex chromosome in additional eublepharid species with GSD to reconstruct evolutionary history of sex determination in this lineage. The genera *Coleonyx* and *Goniurosaurus* are phylogenetically separated by the ESD lineage containing *Eublepharis macularius* and *Hemitheconyx caudicinctus*. According to the ancestral ESD hypothesis, sex chromosomes should have evolved independently in the ancestor of the genera *Goniurosaurus* and *Coleonyx*. Eublepharids are an ideal group to test this crucial prediction of this hypothesis.

Emerging molecular and molecular cytogenetic evidence supports that geckos evolved sex chromosomes independently multiple times. The male-heterogametic sex chromosomes in the genus *Coleonyx* are partially homologous to GGA1, GGA6 and GGA11 (this study), the XX/XY sex chromosomes of the pygopodid geckos to GGA4q [22], the ZZ/ZW sex chromosomes of the sphaerodactylid genus *Aristelliger* to GGA2 [24] and the ZZ/ZW sex chromosomes of several species of the genus *Paroedura* to GGA4p and GGA15 [16]. The sex chromosomes are homologous to the GGAZ syntenic block, both in the phyllodactylid *Phyllodactylus wirshingi* [23] and the gekkonid *Gekko hokouensis* [54], two widely diverged species phylogenetically separated by lineages with other sex determination systems [16,17,18,19,20,21,22,23]. Therefore, it seems that the ancestors of these two species independently co-opted the same region for the role of sex chromosomes rather than that sex chromosomes were in these two lineages inherited from their common ancestor. 

Despite the overall variability in sex determination in geckos, it seems that sex chromosomes in several gekkotan lineages are old and stable. The legless geckos from the family Pygopodidae demonstrate a long-term stability of sex chromosomes for 32–72 MY [22]. Several species of the genus *Paroedura* (family Gekkonidae) demonstrate homologous sex chromosomes for 60–92 MY, with a more recent turnover of an inner clade to a novel GSD system [16]. In addition, conserved sex chromosomes were also recently described in the sphaerodactylid genus *Aristelliger* [24]. The genus *Coleonyx* with sex chromosomes conserved for around 34 million years as revealed here further demonstrate that several gekkotan subclades have evolutionary stable sex chromosomes. In summary, the current evidence suggests that several gecko sublineages have independently evolved sex chromosomes stable for a long time, which contrasts with the assumed frequent turnovers of sex determination systems in this group. Nevertheless, we must stress that the data enabling testing homology of the sex determination systems across the gecko megaphylogeny are still very scarce. Our limited knowledge on the sex determination systems in geckos prohibits a safe conclusion on the ancestral sex determination in this lineage, but also in squamate reptiles. Future studies should focus on revealing the sex determination systems in additional lineages of geckos by carefully controlled incubation experiments in a wide range of temperatures to uncover ESD and by next generation sequencing methodologies (e.g., DNAseq, RNAseq, RADseq) to identify the sex chromosome gene content, to test the homology of sex chromosomes in a wider phylogenetic spectrum.

## 4. Materials and Methods 

### 4.1. Samples and Species Verification

Blood samples were collected from both sexes in 12 species of eublepharid geckos and in two species of phyllodactylid and one species of sphaerodactylid geckos (Table S1). All specimen originated from legal imports kept in our animal facility and/or were captive bred animals available in pet trade. The procedures on animal handling were approved from the Ethical Committee of the Faculty of Science, Charles University (permission No. 29555/2006-30).

Total DNA was isolated using the DNeasy Blood and Tissue Kit (Qiagen, Valencia, CA, USA). The taxon identification was based on taxonomical characters of external morphology. For all specimens, we provide the partial sequence of the mitochondrial gene cytochrome c oxidase I (COI), as a genetic identity for future comparative studies. In details, we amplified the standard DNA barcoding region (664bp) of COI gene following a standard PCR protocol [55] with primers optimized for reptiles [56]. The PCR products were purified and sequenced bi-directionally by Macrogen (Seoul, Korea). The COI sequences were subsequently trimmed in FinchTV and compared to sequences deposited in public databases by BLASTn [57] to verify the taxon assessment. All sequences were deposited in GenBank database, under the accession numbers MW326645- MW326657.

### 4.2. DNA-seq and Coverage Analysis

Genomic DNA from one male and one female of *Coleonyx elegans* were sequenced at high coverage by Novogene (Cambridge, UK) on Illumina HiSeq2500 platform with 350bp pair-end option (DNA-seq). The Illumina adapters and low-quality bases were trimmed by Trimmomatic v0.39 [58] with default parameters. Reads shorter than 50 bp were discarded from further analysis. The quality of the trimmed reads was checked in FASTQC [59]. The raw Illumina reads are deposited in GenBank database, under the BioProject PRJNA682555.

The trimmed Illumina reads from the male and the female specimen were independently mapped with Geneious Prime [45] to a reference dataset consisting of 170,981 exons, extracted from the *Gekko japonicus* genome project [46]. The average read coverage per gene was calculated in each specimen after filtering all exons with unexpectedly high or low coverage (3-fold difference from the mode coverage). Subsequently, we calculated the ratio of male to female (m/f) read coverage for each gene normalized to the total number of assembled reads per specimen (see [60,61]) (Table S2).

The depth coverage of the reads from DNA-seq in Illumina HiSeq platforms is expected to be proportional to the amount of DNA molecules used for library preparation and sequencing. Therefore, after normalization for differences in concentration of the DNA sample, the X-specific loci are expected to have half read coverage in males in comparison to females in XX/XY sex determination systems with degenerated Y chromosomes, while autosomal, pseudoautosomal and poorly differentiated loci should have equal read coverage in both sexes (see [60,61]).

The chromosome level assemblies of the green anole *Anolis carolinensis* [62], the common wall lizard *Podarcis muralis* [63] and the chicken *Gallus gallus* [64] were used for genome-wide cross species comparisons of the homology of sex chromosomes (Table S2).

### 4.3. Validation of X-Specific Genes by qPCR in C. elegans and Test of Homology to Other Geckos

We used a qPCR method to compare the relative gene copy variation between the male and female genome described in detail in Rovatsos and co-authors [16,22,65,66,67], in order to validate the X-specificity of the genes revealed from the comparative coverage analysis (Table S3). In similar approach as for the comparative genome coverage, the XY males have double copies of X-specific genes compared to the XX females. Such difference between male and female genomes in the copies of the X-specific genes can be estimated by qPCR. Therefore, the male to female ratio (r) in gene copy number is expected to be 0.5 for the X-specific genes, 1.0 for autosomal or pseudoautosomal autosomal genes, and 2.0 for the Z-specific genes.

Primers specific for 10 X-specific genes of *C. elegans* (Table S3)*,* selected from the comparative coverage analysis, were designed by Primer-Blast Software [68] using Primer3 [69]. The gene *noct* was used as autosomal controls and the gene *mecom* was used for the normalization of the qPCR values. The primers for the amplification of the genes *noct* and *mecom* were previously published by Rovatsos and co-authors [16].

In addition, six X-specific genes of *C. elegans* were subsequently tested by qPCR to explore the homology of sex chromosomes across 11 additional species from the family Eublepharidae, two species from the family Phyllodactylidae and one species from the family Sphaerodactylidae (Table S3).

## Supplementary Materials

The following are available online at https://www.mdpi.com/2075-1729/10/12/342/s1, Table S1: List of geckos, per species and sex, used in the current study, Table S2: Read coverage analysis in *Coleonyx elegans*: the average coverage per gene in male and female, as well as the normalized male to female coverage ratio are presented. In addition, the homology of examined genes to the genes of *Anolis carolinensis*, *Podarcis muralis* and *Gallus gallus* is presented, Table S3: List of primers and relative male to female gene dose ratios, estimated from qPCR quantification values, Table S4: Genes involved in gonad development and differentiation from the genomic regions of GGA1, GGA6 and GGA11.
